# Dual-graph knowledge distillation for few-shot class-incremental microorganism recognition

**DOI:** 10.3389/fmicb.2026.1791871

**Published:** 2026-03-09

**Authors:** Sihang Xu, Yangfan Hu, Yinuo Zhang, Liwen Chen, Yimin Yin

**Affiliations:** 1 School of Computer, Hunan First Normal University, Changsha, China; 2 School of Computer Science and Mathematics, Fujian University of Technology, Fuzhou, China; 3 College of Meteorology and Oceanography, National University of Defense Technology, Changsha, China; 4 Rocket Force University of Engineering, Xi'an, China; 5 School of Mathematics and Statistics, Hunan First Normal University, Changsha, China

**Keywords:** contrastive-inspired representation learning, environmental microorganism recognition, few-shot class-incremental learning, graph-based knowledge distillation, prototype rectification

## Abstract

Environmental microorganism recognition from microscopic images is crucial for environmental monitoring and ecological analysis. In practical scenarios, microorganism categories often evolve over time, and newly emerging classes usually have only a few labeled samples due to high annotation costs. This combination naturally gives rise to the few-shot class-incremental learning (FSCIL) problem. FSCIL requires models to incrementally learn new classes under severe data scarcity while effectively retaining knowledge of previously learned ones. In this work, we propose a unified FSCIL framework for environmental microorganism recognition. The proposed method is composed of three complementary components. First, a contrastive-inspired fine-grained representation learning strategy is introduced in the base session. This strategy enhances intra-class compactness by mining prediction-consistent augmented samples, without introducing explicit contrastive losses. Second, a prototype rectification mechanism is designed to stabilize the representations of incremental classes by leveraging semantic structures learned from base classes. Third, a dual-graph knowledge distillation framework is proposed to preserve both instance-level and class-level relational knowledge during incremental learning. This process is guided by a teacher model updated via exponential moving average. Experiments conducted on the EMDS-7 dataset demonstrate the effectiveness of the proposed approach. Compared with state-of-the-art FSCIL methods, our method achieves the highest average accuracy of 78.19% and maintains the best final-session accuracy of 65.36%. Meanwhile, strong base-session performance is consistently preserved. These results indicate that the proposed framework effectively mitigates catastrophic forgetting and enables robust adaptation to new microorganism categories in real-world incremental recognition scenarios.

## 1 Introduction

Environmental microbiome research plays a crucial role in understanding ecological processes, environmental health, and microbial diversity. In recent years, advances in microscopy imaging techniques have enabled detailed observation of microbial morphology and structural characteristics in environmental samples. These advances have led to the construction of microscopy-based environmental microorganism image datasets (University VIII, 2025). Compared with sequencing-based approaches, microscopy images provide complementary visual information. They are particularly valuable for morphology-driven microorganism classification and analysis (Mermans et al., 2023).

However, environmental microorganism datasets derived from microscopy images pose substantial challenges for automated classification. Such data typically exhibit complex visual patterns and fine-grained morphological variations. At the same time, pronounced intra-class variability and strong inter-class similarity are commonly observed among different microorganism categories. In addition, microorganisms often appear as small or weakly visible objects embedded in cluttered backgrounds. These characteristics make robust image-based classification particularly challenging (Giner et al., 2016; Yin et al., 2025).

Beyond these inherent visual challenges, environmental microorganism classification tasks are rarely static in practical applications. They often evolve over time as research objectives or annotation criteria are refined, requiring classification systems to continuously extend their recognition scope by incorporating additional microorganism categories. Meanwhile, stable identification performance for previously learned categories must be maintained. This requirement naturally gives rise to the class-incremental learning (CIL) paradigm, which focuses on enabling models to learn new categories sequentially without retraining from scratch.

In addition to class incrementality, environmental microorganism image data are also characterized by severe data scarcity at the category level. Due to the high cost of sample collection and the reliance on expert knowledge for accurate annotation, newly introduced microorganism categories are often represented by only a small number of labeled microscopy images. This data-limited scenario motivates the few-shot learning (FSL) paradigm, which aims to enable effective learning of new categories from very limited labeled samples. When class incrementality and data scarcity coexist, the problem becomes few-shot class-incremental learning (FSCIL) (Tao et al., 2020). In this setting, models are required to rapidly adapt to new microorganism categories from only a few labeled images while effectively retaining knowledge of previously learned categories. Although recent FSCIL methods have achieved encouraging results on general computer vision benchmarks, their performance often degrades when applied to environmental microorganism microscopy images. This limitation can be attributed to domain-specific factors, including complex morphological variability, high visual similarity between different microorganism types, and variability introduced during image acquisition and preprocessing.

**Figure 1:**
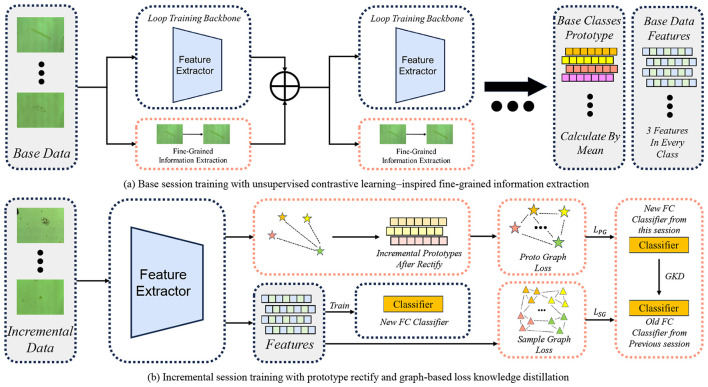
Overall framework of the proposed method for few-shot class-incremental learning. **(a)** Base session training: the feature extractor is optimized on base-class data with the proposed fine-grained information extraction module, where prediction-consistent locally augmented samples are iteratively mined to enhance intra-class representation compactness, without introducing explicit contrastive objectives. Base class prototypes and representative features are then computed for subsequent incremental learning. **(b)** Incremental session training: class prototypes of incremental classes are first rectified using base-class knowledge, followed by graph-based knowledge distillation. Both prototype-level graph loss and sample-level graph loss are employed to transfer relational knowledge from previous sessions, enabling stable adaptation to incremental classes while alleviating catastrophic forgetting.

To address these challenges, we propose a novel few-shot class-incremental classification algorithm tailored for microscopy-based environmental microorganism image data. The proposed approach is designed to balance model plasticity and stability under severe data scarcity, enabling robust learning of newly introduced microorganism categories while mitigating catastrophic forgetting. In particular, the base-session training adopts a consistency-based fine-grained representation learning strategy, which is conceptually inspired by contrastive learning but does not employ explicit contrastive losses or negative sample mining. Extensive experiments on a representative environmental microorganism microscopy dataset demonstrate the effectiveness of the proposed approach across various incremental learning settings. These results highlight its robustness and practical applicability for real-world environmental microorganism classification tasks. An overview of the overall learning paradigm, including the base session and incremental sessions, is illustrated in Figure 1.

## 2 Related work

### 2.1 Few-shot learning

FSL aims to recognize novel categories from only a few labeled samples by leveraging prior knowledge learned from base classes. Early research in this area has primarily focused on metric-based approaches, which learn an embedding space where samples can be classified by measuring distances to class prototypes or support examples (Wang et al., 2020). Representative methods include prototypical networks, matching networks, and relation networks, where classification is performed via nearest-neighbor or similarity-based inference in the learned feature space (Vinyals et al., 2016; Snell et al., 2017; Sung et al., 2018). Complementary to metric-based formulations, another line of research explores optimization-based methods that aim to learn model initialization or update strategies capable of rapidly adapting to new tasks with limited supervision. Meta-learning approaches such as MAML and its variants fall into this category, enabling fast parameter adaptation through gradient-based updates (Li et al., 2017). Despite their effectiveness on standard few-shot benchmarks, these methods often require task-specific fine-tuning and may suffer from instability when extended to sequential learning scenarios.

More recent studies have further explored data-centric solutions to alleviate the few-shot bottleneck. In particular, generative and augmentation-based methods have been proposed to synthesize additional samples or enrich feature distributions for incremental classes, thereby improving generalization under extremely low-shot conditions (Wang et al., 2018). However, most existing few-shot learning methods are developed under a static task assumption, where all categories are available simultaneously during training. As a result, these approaches do not explicitly consider the continual introduction of new classes, limiting their applicability to realistic incremental learning scenarios (Hospedales et al., 2021). Overall, while few-shot learning has achieved significant progress in learning from limited data, most existing approaches are not directly applicable to class-incremental settings, motivating the development of more robust frameworks such as few-shot class-incremental learning. However, most few-shot learning methods are designed for static task settings and do not account for continual class expansion, making them insufficient for scenarios where new microorganism categories are introduced sequentially under severe data scarcity.

### 2.2 Class-incremental learning

CIL studies how to continuously learn a sequence of tasks where incremental classes are introduced over time, while maintaining performance on previously learned classes without accessing all past data (Zhou et al., 2023). A central challenge in CIL is catastrophic forgetting, where the model rapidly degrades on old classes due to distribution shift and parameter interference during incremental updates. To address this issue, existing CIL methods can be broadly categorized into several representative paradigms, including replay-based approaches, regularization-based approaches, and parameter isolation or expansion strategies (Masana et al., 2022). Replay-based methods alleviate forgetting by revisiting previous knowledge, either through storing a small subset of old samples (exemplar rehearsal) or synthesizing pseudo-samples using generative models (Shin et al., 2017). Representative techniques maintain a memory buffer to approximate the past data distribution, enabling the model to jointly optimize on old and incremental classes while balancing memory size and computational cost (Rebuffi et al., 2017). In contrast, regularization-based methods constrain parameter updates to preserve knowledge important for old tasks, such as elastic weight consolidation and related importance-weighted penalties (Kirkpatrick et al., 2017). Another complementary line of work performs knowledge distillation between the current model and a frozen previous model to preserve old decision boundaries, typically by matching logits or intermediate representations (Li and Hoiem, 2017).

Beyond these mainstream paradigms, several studies explore architecture-level solutions to mitigate catastrophic forgetting. These methods include dynamically expanding networks, isolating parameters for different tasks, or learning task-specific adapters, which reduce interference at the cost of additional parameters and architectural complexity (Rusu et al., 2016; Zhu et al., 2021). More recently, attention has been drawn to the stability of the learned representation space, as feature drift across incremental sessions can severely harm exemplar-free incremental learning. Despite these advances, standard CIL methods typically assume sufficient labeled data for incremental classes and do not explicitly address the extreme low-shot regime. This limitation motivates the integration of few-shot learning and class-incremental learning, giving rise to more challenging settings such as few-shot class-incremental learning and the need for more robust knowledge transfer mechanisms. Despite their effectiveness in mitigating catastrophic forgetting, most CIL methods assume sufficient labeled data for incremental classes. This assumption is often violated in environmental microorganism recognition, where new categories typically contain only a few annotated samples, limiting the applicability of standard CIL techniques.

### 2.3 Few-shot class-incremental learning

FSCIL extends class-incremental learning to a more challenging setting where only a few labeled samples are available for each newly introduced class (Tao et al., 2020). In this scenario, models must continuously incorporate incremental classes from extremely limited data while preserving performance on previously learned ones, making FSCIL particularly susceptible to both catastrophic forgetting and overfitting (Zhang et al., 2025c; Tian et al., 2024). Early studies in FSCIL primarily adopt prototype-based paradigms, in which class representations are summarized by feature prototypes computed from limited samples (Snell et al., 2017; Zhang et al., 2025a,d). Representative approaches incrementally update classifiers by estimating class prototypes through feature averaging, thereby avoiding extensive parameter updates and alleviating forgetting. To further enhance stability, some methods constrain the update process by freezing the feature extractor after the base session, effectively treating FSCIL as a classifier adaptation problem on a fixed embedding space (Zhang et al., 2021).

Despite their effectiveness, prototype-based strategies alone often struggle to balance stability and plasticity under severe data scarcity. To address this limitation, several studies introduce knowledge distillation mechanisms into FSCIL (Cheraghian et al., 2021; Zhang et al., 2025b). These methods typically distill knowledge from a frozen model trained in previous sessions to the current model, aligning logits or intermediate representations to preserve base-class information. More recent works further extend this idea by exploiting multiple teachers, class-aware distillation, or relational constraints to enhance knowledge transfer under few-shot conditions (Dong et al., 2021; Zhao et al., 2023). Overall, FSCIL remains a highly challenging problem due to the combined difficulties of data scarcity and continual learning. This challenge motivates the development of more robust representation learning and knowledge transfer mechanisms that can more effectively leverage base-session information to support long-term incremental generalization. Although existing FSCIL methods have shown promising results on generic benchmarks, their performance often degrades in fine-grained domains such as environmental microorganism recognition. In particular, limited sample availability, subtle inter-class differences, and long-term representation drift pose additional challenges that are not sufficiently addressed by current approaches.

### 2.4 Environmental microorganism recognition

Environmental microorganism recognition aims to automatically identify microorganism categories from microscopic images collected in natural or industrial environments. Compared with generic visual recognition tasks, this problem is particularly challenging due to large intra-class variations, subtle inter-class differences, and significant visual noise caused by imaging conditions, illumination changes, and complex backgrounds (Kulwa et al., 2022; Liang et al., 2021). Early research efforts primarily relied on handcrafted features combined with classical machine learning classifiers, including texture descriptors, shape statistics, and color-based features, to characterize microorganisms (Yang et al., 2014). While these approaches achieved limited success, their performance was constrained by the representational capacity of manually designed features. With the rapid development of deep learning, convolutional neural networks (CNNs) have become the dominant paradigm for microorganism recognition. By learning discriminative representations directly from raw image data, CNN-based methods have significantly improved recognition accuracy and robustness compared with traditional approaches (Khasim et al., 2024).

Despite these advances, most existing microorganism recognition methods are developed under a static closed-set classification assumption, where all microorganism categories are predefined and remain unchanged during training (Zhao et al., 2022a,b). However, in practical environmental monitoring scenarios, microorganism distributions are inherently dynamic, and new categories may continuously emerge, often accompanied by only a few annotated samples. Under such conditions, retraining models from scratch is both inefficient and impractical. These limitations motivate the adoption of continual and incremental learning paradigms for environmental microorganism recognition. In particular, combining few-shot learning with class-incremental learning provides a more realistic framework, enabling models to efficiently incorporate novel microorganism categories while retaining knowledge of previously learned classes. Most existing microorganism recognition methods focus on closed-set classification and lack mechanisms to accommodate both continual class evolution and extreme data scarcity, which are common in real-world environmental monitoring scenarios.

## 3 Proposed method

In this section, we present a unified framework for few-shot class-incremental learning that is designed to maintain representation stability under sequential class expansion and severe data scarcity. Rather than addressing incremental learning challenges in isolation, our method explicitly targets complementary sources of instability that arise at different semantic levels during FSCIL, including instance-level feature variance, unreliable class prototypes estimated from few samples, and progressive distortion of relational geometry across sessions. Based on this perspective, the proposed framework integrates three tightly coupled components that operate in different stages of learning. Specifically, we first enhance fine-grained representation quality in the base session through a consistency-guided feature learning strategy, establishing a compact and discriminative embedding space. This strategy is conceptually inspired by contrastive learning in terms of representation invariance, but does not employ explicit contrastive loss functions or positive–negative pair construction. Building upon this foundation, we introduce a prototype rectification mechanism to stabilize the representations of incremental classes by leveraging well-established base-class semantics. Finally, we preserve structural knowledge during incremental learning via a graph-based knowledge distillation framework that jointly constrains instance-level and class-level relationships. Together, these components form a coherent learning paradigm that progressively builds robust representations and effectively mitigates catastrophic forgetting in FSCIL settings.

### 3.1 Contrastive-inspired fine-grained representation learning

**Figure 2:**
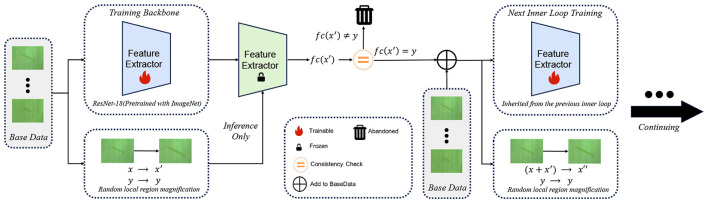
Overview of the unsupervised contrastive learning–inspired fine-grained information extraction framework in the base session. Each base session training epoch is divided into multiple inner-loop. The feature extractor is first trained on the original labeled data, while locally magnified samples are generated via stochastic augmentation. After each inner-loop, the augmented samples are forwarded through the model in an inference-only manner, and only those with consistent predictions are retained as pseudo-samples and merged into the next inner-loop's training set. This iterative augmentation-and-filtering process progressively enriches the training data with fine-grained and semantically consistent samples without introducing explicit contrastive losses.

In FSCIL, the base session plays a pivotal role in shaping the global structure of the feature space. The quality of representations learned at this stage directly determines the stability and generalization capability of subsequent incremental sessions. However, conventional supervised training primarily emphasizes coarse inter-class separability, while fine-grained intra-class variations are often insufficiently explored, leading to suboptimal representation robustness. Figure 2 provides an overview of the proposed contrastive-inspired fine-grained representation learning framework employed during the base session.

Inspired by unsupervised contrastive learning, we propose a fine-grained information extraction strategy that enhances intra-class compactness by leveraging prediction consistency under strong data augmentations. Instead of explicitly introducing contrastive objectives or negative sample mining, our method implicitly encourages representation alignment through a consistency-based pseudo-sample selection mechanism.

Formally, let $D={\left\{\left(x_{i},y_{i}\right)\right\}}_{i=1}^{N}$ denote the labeled training set in the base session, where $x_{i}\in {\mathbb{R}}^{H\times W\times C}$ and *y*_*i*_ ∈ {1, …, *C*_*b*_}. We define a stochastic data augmentation operator T(·) that generates an augmented view ${\tilde{x}}_{i}=T\left(x_{i}\right)$. Given a neural network *f*_θ_(·), the predicted label of the augmented sample is obtained as


$$\begin{array}{l} {ŷ}_{i}=\arg\underset{c}{\max}f_{\theta }{\left({\tilde{x}}_{i}\right)}_{c}. \end{array}$$ (1)

A pseudo-sample $\left({\tilde{x}}_{i},y_{i}\right)$ is retained only if the prediction remains consistent with the original ground-truth label, i.e.,


$$\begin{array}{l} {ŷ}_{i}=y_{i}. \end{array}$$ (2)

The retained pseudo-samples are progressively merged with the original training set and used for subsequent optimization cycles within the same training epoch. This iterative augmentation-and-selection process gradually enriches the training distribution with semantically reliable samples.

From a theoretical perspective, unsupervised contrastive learning aims to maximize the agreement between different augmented views of the same instance. A representative contrastive objective can be expressed as


$$\begin{array}{l} L_{\text{CL}}=-𝔼\left[\log\frac{\exp\left(\text{sim}\left(z_{i},z_{i}^{+}\right)/\tau \right)}{\sum_{j}\exp\left(\text{sim}\left(z_{i},z_{j}\right)/\tau \right)}\right], \end{array}$$ (3)

where *z*_*i*_ = *f*_θ_(*x*_*i*_) and $z_{i}^{+}=f_{\theta }\left(T\left(x_{i}\right)\right)$ denote the representations of the original and augmented samples, respectively.

Although no explicit contrastive loss is imposed in our formulation, the prediction consistency constraint


$$\begin{array}{l} \arg\max f_{\theta }\left(x_{i}\right)=\arg\max f_{\theta }\left(T\left(x_{i}\right)\right) \end{array}$$ (4)

implicitly enforces that the similarity between positive pairs exceeds that of mismatched pairs, i.e.,


$$\begin{array}{l} \text{sim}\left(z_{i},z_{i}^{+}\right)\geq \text{sim}\left(z_{i},z_{j}\right),\;\forall j\neq i. \end{array}$$ (5)

As a result, the proposed consistency-based filtering mechanism can be interpreted as a lower-bound approximation of contrastive representation alignment.

Furthermore, let $P_{c}$ denote the set of retained pseudo-samples for class *c*. Under the consistency constraint, minimizing the cross-entropy loss over the augmented training set D∪P leads to enhanced intra-class compactness:


$$\begin{array}{l} {𝔼}_{x_{i},x_{j}\in P_{c}}||f_{\theta }\left(x_{i}\right)-f_{\theta }\left(x_{j}\right){||}_{2}^{2}↓. \end{array}$$ (6)

This is because samples that violate class decision boundaries are systematically filtered out, while training on multiple consistent augmented views encourages feature embeddings to contract around class prototypes, analogous to positive-pair attraction in contrastive learning.

In summary, the proposed method introduces an unsupervised contrastive learning–inspired pseudo-sample mining strategy for fine-grained representation extraction. By enforcing prediction consistency under strong data augmentations, the model progressively learns invariant yet discriminative features without relying on explicit contrastive losses. This process significantly improves intra-class compactness and establishes a robust foundation for prototype estimation and knowledge transfer in subsequent incremental learning stages.

### 3.2 Prototype rectification for incremental class adaptation

In FSCIL, class prototypes serve as compact representations of category-level semantics and play a crucial role in classification and knowledge transfer. However, prototypes of incremental classes are typically estimated from extremely limited samples, making them highly susceptible to noise and sampling bias. Directly incorporating such unreliable prototypes may lead to representation drift and performance degradation.

**Figure 3:**
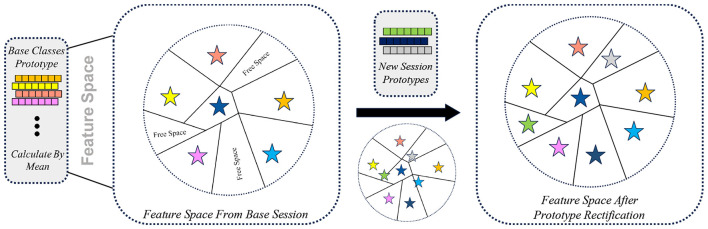
Illustration of prototype rectification in the feature space during incremental learning. **(Left)** the feature space learned in the base session, where class prototypes are computed as the mean embeddings and leave unused feature space for future classes. **(Middle)** prototypes introduced in a new session are initially embedded close to existing regions, which may cause semantic overlap. **(Right)** after prototype rectification, new class prototypes are adaptively adjusted toward semantically consistent regions while preserving the relative structure of base classes, resulting in a more balanced and discriminative feature space.

To address this issue, we propose a prototype rectification mechanism that leverages well-established base class prototypes to regularize and refine newly learned incremental prototypes. The core intuition is that base class prototypes, learned from abundant data, provide a stable semantic reference space that can guide the adaptation of novel class representations Figure 3.

Formally, let $\text{P}=\left[{\text{p}}_{1},\ldots ,{\text{p}}_{C}\right]\in {\mathbb{R}}^{C\times d}$ denote the set of class prototypes, where the first *C*_*b*_ prototypes correspond to base classes and the remaining *C*_*n*_ = *C*−*C*_*b*_ prototypes correspond to incremental classes. Let ${\text{p}}_{i}^{\left(s\right)}$ denote the prototype of the *i*-th incremental class extracted by the student model.

To rectify the incremental prototypes, we first compute the cosine similarity between each incremental prototype and all base class prototypes:


$$\begin{array}{l} {\text{s}}_{i,j}=\frac{{\text{p}}_{i}^{\left(s\right)}\cdot {\text{p}}_{j}^{\left(b\right)}}{||{\text{p}}_{i}^{\left(s\right)}{||}_{2}||{\text{p}}_{j}^{\left(b\right)}{||}_{2}},\;j\in \left\{1,\ldots ,C_{b}\right\}. \end{array}$$ (7)

For each incremental prototype, we identify the most semantically related base class prototype:


$$\begin{array}{l} j^{*}=\arg\underset{j}{\max}{\text{s}}_{i,j}. \end{array}$$ (8)

The rectified prototype is then obtained via a convex combination:


$$\begin{array}{l} {\tilde{\text{p}}}_{i}=\left(1-\beta \right){\text{p}}_{i}^{\left(s\right)}+\beta {\text{p}}_{j^{*}}^{\left(b\right)}, \end{array}$$ (9)

where β ∈ [0, 1] controls the strength of prototype rectification.

The rectified prototypes are subsequently used to replace the original incremental prototypes during training, providing a smoother and more stable representation transition between base and incremental classes. As a result, the proposed rectification strategy is most effective when the base session covers a sufficiently diverse set of classes to establish a meaningful feature manifold, and when incremental classes lie within or near this learned representation space.

### 3.3 Graph-based knowledge distillation

**Figure 4:**
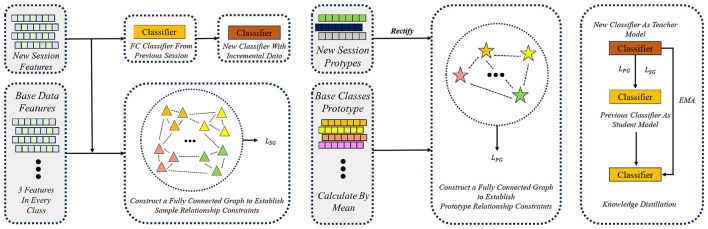
Dual-graph knowledge distillation with sample-level and prototype-level relational losses. During incremental learning, a student model is optimized under the guidance of a teacher model updated via exponential moving average (EMA). To preserve previously learned relational knowledge, two complementary graph-based distillation losses are introduced. (1) Sample-level graph loss enforces consistency between the student and teacher by aligning pairwise similarity graphs constructed from randomly sampled features, thereby preserving instance-wise relational structures. (2) Prototype-level graph loss constrains the relational geometry between incremental-class prototypes and base-class prototypes, ensuring that newly learned classes are embedded in a semantically consistent manner. By jointly distilling both instance-level and class-level relational knowledge, the proposed dual-graph distillation framework effectively mitigates catastrophic forgetting while enabling stable adaptation to new classes.

While prototype rectification stabilizes the class-level representations of newly introduced categories, it does not explicitly preserve the relational knowledge among samples and classes learned in previous sessions. In class-incremental learning, such relational structures encode high-order information about the data manifold and inter-class geometry, which are crucial for mitigating catastrophic forgetting. To address this limitation, we propose a graph-based knowledge distillation framework that transfers relational knowledge from a teacher model to the student model during incremental training. Figure 4 illustrates how prototype rectification reshapes the feature space, while the proposed graph distillation further constrains relational consistency across sessions.

The proposed graph-based distillation framework incorporates two complementary relational constraints that operate at different semantic levels. The sample-level graph distillation focuses on preserving instance-wise relational structures, which capture local geometry and fine-grained similarity relationships among individual samples. In contrast, the prototype-level graph distillation targets class-level relational geometry, explicitly maintaining the relative positioning between incremental-class prototypes and base-class prototypes. Together, these two constraints jointly regularize both local and global aspects of the representation space during incremental learning.

At the beginning of each incremental session, the teacher model is initialized as an exact copy of the student model obtained from the previous session, thereby anchoring the teacher to the knowledge learned so far. During training within the current session, the teacher parameters are not updated by back-propagation. Instead, the teacher is updated using an exponential moving average (EMA) of the student parameters:


$$\begin{array}{l} \theta^{\left(t\right)}\leftarrow m\theta^{\left(t\right)}+\left(1-m\right)\theta^{\left(s\right)}, \end{array}$$ (10)

where θ^(*s*)^ and θ^(*t*)^ denote the parameters of the student and teacher models, respectively, and *m*∈[0, 1) is the momentum coefficient. This design allows the teacher to remain anchored to historical knowledge while providing a temporally smoothed reference that reduces noise caused by few-shot updates.

Let


$${\text{F}}^{\left(s\right)}={\left\{{\text{f}}_{i}^{\left(s\right)}\right\}}_{i=1}^{N},\;{\text{F}}^{\left(t\right)}={\left\{{\text{f}}_{i}^{\left(t\right)}\right\}}_{i=1}^{N}$$

denote the feature representations of *N* sampled instances extracted by the student and teacher models, respectively. To capture instance-level relational structures, we construct similarity graphs based on pairwise cosine similarities in the feature space. The student and teacher similarity matrices are defined as


$$\begin{array}{l} {\text{G}}^{\left(s\right)}=\text{cos}\left({\text{F}}^{\left(s\right)},{\text{F}}^{\left(s\right)}\right),\;{\text{G}}^{\left(t\right)}=\text{cos}\left({\text{F}}^{\left(t\right)},{\text{F}}^{\left(t\right)}\right). \end{array}$$ (11)

The instance-level graph distillation loss is formulated as


$$\begin{array}{l} L_{\text{inst}}=||{\text{G}}^{\left(s\right)}-{\text{G}}^{\left(t\right)}{||}_{F}^{2}. \end{array}$$ (12)

Beyond instance-level relationships, we further distill class-level structural knowledge using class prototypes. Let


$${\text{P}}^{\left(s\right)}={\left\{{\text{p}}_{c}^{\left(s\right)}\right\}}_{c=1}^{C},\;{\text{P}}^{\left(t\right)}={\left\{{\text{p}}_{c}^{\left(t\right)}\right\}}_{c=1}^{C}$$

denote the class prototypes produced by the student and teacher models, respectively. Let **P**_*b*_ and **P**_*n*_ denote the prototypes of base classes and incremental classes. The prototype-level relation graphs are defined as


$$\begin{array}{l} {\text{H}}^{\left(s\right)}=\text{cos}\left({\text{P}}_{n}^{\left(s\right)},{\text{P}}_{b}^{\left(s\right)}\right),\;{\text{H}}^{\left(t\right)}=\text{cos}\left({\text{P}}_{n}^{\left(t\right)},{\text{P}}_{b}^{\left(t\right)}\right). \end{array}$$ (13)

The corresponding prototype-level graph distillation loss is given by


$$\begin{array}{l} L_{\text{proto}}=||{\text{H}}^{\left(s\right)}-{\text{H}}^{\left(t\right)}{||}_{F}^{2}. \end{array}$$ (14)

The final training objective integrates standard classification supervision with the proposed graph-based distillation losses:


$$\begin{array}{l} L=L_{\text{CE}}+\alpha L_{\text{inst}}+\gamma L_{\text{proto}}, \end{array}$$ (15)

where $L_{\text{CE}}$ denotes the cross-entropy loss, and α and γ control the contributions of instance-level and prototype-level graph distillation, respectively. To prevent classification supervision from overwhelming relational constraints under few-shot conditions, a warm-up strategy is adopted in which graph-based distillation dominates the early training stages, allowing the student model to first align its relational structures with the teacher before jointly optimizing classification performance.

## 4 Experiment

### 4.1 Dataset and metric

**Figure 5:**
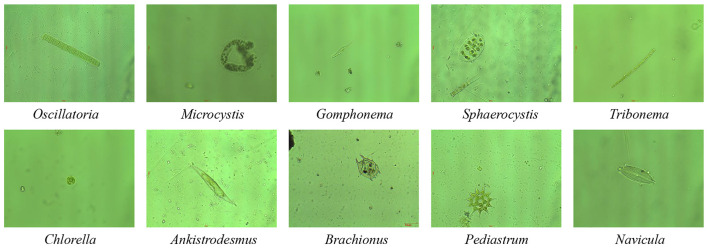
Examples in the EMDS-7 dataset.

All experiments are conducted on the EMDS-7 dataset, a publicly available environmental microorganism image dataset designed for microscopic image analysis (Yang et al., 2023). EMDS-7 contains microscopic images collected from lakes and rivers in Shenyang, China, and covers a total of 41 microorganism categories annotated by domain experts. The dataset exhibits fine-grained visual characteristics with substantial intra-class variability and high inter-class similarity, which makes it particularly challenging under few-shot and incremental learning settings.Representative microscopy images from the EMDS-7 dataset are shown in Figure 5, highlighting the fine-grained visual characteristics and inter-class similarity.

Following the standard protocol of few-shot class-incremental learning (FSCIL), the dataset is reorganized into a base session and a sequence of incremental sessions. Specifically, 20 classes are selected as base classes and are trained with relatively sufficient labeled samples to establish an initial representation space. The remaining 21 classes are introduced incrementally under a 3-way 3-shot setting, where each incremental session adds three novel classes with only three labeled samples per class. After each session, the model is evaluated on all classes encountered so far, without access to training data from previous sessions, reflecting realistic scenarios where annotated microorganism samples are scarce and new categories emerge over time.

To evaluate performance throughout the incremental learning process, we adopt three commonly used metrics in FSCIL. Average Accuracy (AA) measures the mean classification accuracy over all sessions, reflecting the overall performance across the entire learning trajectory. Let *S* denote the total number of sessions and Acc_*s*_ denote the classification accuracy after session *s*, then AA is defined as


$$\begin{array}{l} \text{AA}=\frac{1}{S}\overset{S}{\underset{s=1}{\sum }}\text{Ac}{\text{c}}_{s}. \end{array}$$ (16)

Performance Drop (PD) quantifies the degree of catastrophic forgetting by measuring the accuracy degradation from the base session to the final incremental session, defined as


$$\begin{array}{l} \text{PD}=\text{Ac}{\text{c}}_{\text{base}}-\text{Ac}{\text{c}}_{\text{final}}. \end{array}$$ (17)

In addition, we report the classification accuracy at each incremental session to analyze the performance evolution and stability of the model during the incremental learning process.

### 4.2 Implementation details

All experiments are implemented using the PyTorch framework and conducted on a NVIDIA A100 GPU. The random seed is fixed to ensure reproducibility. For all datasets, we adopt a ResNet-18 backbone initialized with ImageNet-pretrained weights, followed by a cosine similarity-based classifier.

During the base session, the model is optimized using stochastic gradient descent with a momentum of 0.9 and a weight decay of 5 × 10^−4^. The initial learning rate is set to 0.1 and decayed by a factor of 0.1 using a step-based scheduler. The base session is trained for 100 epochs with a batch size of 1,024. To enhance representation robustness, we employ a multi-cycle pseudo-sample augmentation strategy, where each training epoch consists of 10 augmentation cycles. In each cycle, only pseudo-samples whose predictions remain consistent with their original labels are retained and merged with the original training set.

After completing the base session, class prototypes are initialized by averaging the feature embeddings of training samples. Incremental learning is then performed using a graph-based knowledge distillation framework, where a frozen teacher model guides the student model. Incremental training is conducted for 20 epochs per session using the same optimizer settings as in the base session.

Prototype rectification is enabled throughout incremental learning. For each newly introduced class, its prototype is rectified by interpolating between the student-estimated prototype and the most semantically similar base class prototype, with the rectification coefficient β set to 0.3. This process stabilizes prototype estimation under extremely limited data.

Unless otherwise specified, all remaining hyperparameters follow standard settings used in few-shot class-incremental learning benchmarks.

### 4.3 Comparison with state of the arts

We compare the proposed method with six representative state-of-the-art FSCIL methods, covering diverse technical paradigms:

- **CEC** (Zhang et al., 2021) adopts a decoupled learning strategy that freezes the backbone network and evolves a graph-based classifier for incremental classes.
- **Limit** (Zhou et al., 2022) introduces a meta-learning framework that synthesizes fake incremental tasks from the base dataset to learn generalizable features.
- **BiDist** (Zhao et al., 2023) proposes a class-aware bilateral distillation mechanism to balance the stability-plasticity dilemma by leveraging two complementary teacher models.
- **TEEN** (Wang et al., 2023) leverages episodic training and task-level normalization to enhance robustness under few-shot incremental settings.
- **PFR** (Pan et al., 2024) is designed for fine-grained scenarios, utilizing pseudo-set frequency refinement to optimize feature spaces.
- **Comp** (Zou et al., 2024) mimics human cognitive processes by decomposing classes into visual primitives and recomposing them for new concepts.
- **CLOSER** (Oh et al., 2024) argues for a more confined feature space to enhance representation transferability.
- **ADBS** (Li et al., 2025) introduces an adaptive debiasing strategy to alleviate classifier bias toward base classes during incremental learning.

The quantitative comparison results on the EMDS-7 dataset are summarized in Table 1. It highlights that the proposed method consistently maintains high accuracy across incremental sessions, resulting in both the highest average accuracy and strong final-session performance among the compared approaches. Beyond numerical performance differences, the proposed framework also differs from existing FSCIL methods in several key methodological aspects. First, unlike approaches such as CEC and Limit that primarily focus on classifier evolution or meta-learning over synthesized tasks, the proposed method explicitly enhances representation quality in the base session through consistency-guided fine-grained feature learning, resulting in a more compact and discriminative embedding space. Second, while several methods rely on fixed or heuristically updated prototypes, the proposed prototype rectification mechanism adaptively stabilizes incremental class representations by leveraging the structural semantics of well-established base classes, which is particularly beneficial under extreme few-shot conditions. Third, compared with distillation-based approaches such as BiDist that mainly align logits or class-level outputs, the proposed dual-graph knowledge distillation framework preserves relational knowledge at both instance and class levels, jointly constraining local feature geometry and global semantic structure. The integration of these complementary components enables the proposed method to better balance plasticity and stability, which explains its superior performance consistency across incremental learning stages.

**Table 1:** Performance comparison under the FSCIL setting on the EMDS-7 dataset.

**Method**	**Venue**	Acc. in each session								**AA↑**	**PD↓**
		**0**	**1**	**2**	**3**	**4**	**5**	**6**	**7**		
CLOSER	ECCV2024	70.9	18.2	17.2	16.3	14.9	14.3	13.3	12.7	22.2	58.2
BiDist	CVPR2023	46.7	44.5	42.0	40.0	38.4	32.1	30.7	28.1	37.8	**18.6**
PFR	PR2024	76.9	75.1	70.1	68.0	67.4	62.0	61.7	56.8	67.2	20.1
Limit	TPAMI2024	80.3	77.2	75.0	70.1	66.6	65.2	61.0	54.3	68.7	26.0
Comp	ICML2024	80.7	77.2	75.8	71.1	69.2	64.3	60.1	57.6	69.5	23.1
CEC	CVPR2021	85.4	82.3	77.1	75.0	74.1	67.4	66.2	60.9	73.5	24.5
TEEN	NIPS2023	88.3	87.1	82.4	**80.1**	75.3	72.0	69.2	62.1	77.1	26.2
ADBS	AAAI2025	90.2	85.8	80.0	78.5	77.1	69.8	**71.1**	64.0	77.1	26.2
Ours	–	**90.58**	**87.14**	**83.23**	79.99	**77.57**	**73.57**	68.08	**65.36**	**78.19**	25.21

Accuracies (%) are reported for each session. AA, Average Accuracy; PD, Performance Drop. The bold values indicate the best performance in each column.

#### 4.3.1 Base session performance

As shown in the Session 0 column of Table 1, the proposed method achieves a base-session accuracy of 90.58%, which is among the highest across all evaluated approaches. The base-session performance is comparable to that of ADBS (90.2%) and higher than several recent FSCIL methods such as TEEN (88.3%), as well as earlier approaches including Limit (80.3%) and CEC (85.4%). These results indicate that the proposed method is able to learn discriminative representations during the base session, which provides a reliable initialization for subsequent incremental learning. This property is particularly relevant for environmental microorganism recognition, where fine-grained morphological differences need to be captured from limited data.

#### 4.3.2 Performance across incremental sessions

Beyond the base session, the proposed method maintains stable performance across all incremental learning stages. Recent methods such as TEEN and ADBS achieve competitive Average Accuracy (AA) scores of 77.1%, benefiting from episodic normalization and adaptive debiasing strategies, respectively. In comparison, the proposed approach attains an AA of 78.19%, resulting in the highest average accuracy among the evaluated methods. Earlier approaches, including BiDist and CLOSER, exhibit more pronounced performance degradation as the number of incremental sessions increases. Overall, the results suggest that the proposed method is effective in maintaining performance consistency under sequential class expansion.

#### 4.3.3 Performance drop and final session accuracy

Table 1 further reports Performance Drop (PD) and final-session accuracy for all methods. BiDist achieves the lowest PD value; however, its absolute accuracy remains relatively low across all sessions. In contrast, TEEN and ADBS present PD values of 26.2%, which are close to that of the proposed method (25.21%). In the final incremental session (Session 7), the proposed approach achieves an accuracy of 65.36%, which is higher than that of ADBS (64.0%) and TEEN (62.1%). These results indicate that the proposed method maintains competitive performance in later incremental stages, where accumulated forgetting effects are typically more pronounced.

## 5 Conclusion

In this work, we investigated the challenging problem of few-shot class-incremental learning (FSCIL) for environmental microorganism recognition, where models must continuously incorporate novel microorganism categories from extremely limited labeled samples while preserving performance on previously learned classes. This problem is particularly challenging due to the coexistence of severe data scarcity, fine-grained visual ambiguity, and the requirement for long-term knowledge retention. To address these challenges, we proposed a unified FSCIL framework that jointly enhances representation robustness, stabilizes class-level semantic information, and preserves structural consistency throughout the incremental learning process. Rather than decomposing FSCIL into isolated subproblems, the proposed framework adopts an integrated learning paradigm that effectively balances model plasticity and stability, thereby alleviating catastrophic forgetting and supporting reliable adaptation to newly introduced microorganism categories.

Extensive experiments conducted on the EMDS-7 environmental microorganism dataset demonstrate the effectiveness of the proposed approach across multiple incremental learning stages. Compared with multiple state-of-the-art FSCIL methods, our framework achieves superior average accuracy and final-session performance while maintaining strong base-session recognition capability. These results indicate that maintaining a stable and discriminative representation space, together with consistent relational structures across sessions, is critical for robust few-shot class-incremental learning in complex microscopy-based recognition scenarios. Beyond quantitative performance gains, the proposed framework also provides a flexible and extensible foundation, which can be readily extended to more diverse environmental datasets and enhanced through label-efficient learning strategies such as self-supervised or semi-supervised incremental learning. In future work, we also plan to investigate lightweight architectures and memory-efficient designs to facilitate real-world deployment in large-scale and long-term environmental monitoring systems.

Despite the encouraging experimental results, the proposed framework also has certain applicability boundaries that merit discussion. First, the effectiveness of the prototype rectification strategy relies on the assumption that the base session contains a sufficiently diverse set of classes to establish a meaningful and well-structured feature space. When incremental classes are largely disjoint from the base-class distribution, the guidance provided by base-class prototypes may become less effective. Second, the graph-based knowledge distillation framework introduces additional computational overhead due to the construction and alignment of relational graphs, which may affect training efficiency when scaling to a large number of classes or samples. Finally, as with most FSCIL methods, the proposed approach assumes a relatively stable data domain across sessions, and significant distribution shifts between base and incremental data may lead to performance degradation.

## Data Availability

The original contributions presented in the study are included in the article/supplementary material, further inquiries can be directed to the corresponding author.
